# Axillary Lymph Node Malignant Melanoma of Unknown Primary Origin: A Case Report From Japan

**DOI:** 10.7759/cureus.71802

**Published:** 2024-10-18

**Authors:** Takeshi Nomura, Gan Muneuchi

**Affiliations:** 1 Public Health, Tokyo Medical and Dental University, Tokyo, JPN; 2 Plastic and Reconstructive Surgery, Osaka Saiseikai Nakatsu Hospital, Osaka, JPN

**Keywords:** axillary lymph nodes dissection, case report, malignant melanoma, melanoma of unknown primary, rare disease

## Abstract

Malignant melanoma of unknown primary origin (MUP) is a rare disease predominantly reported in Caucasian individuals. In this paper, we report a rare case of MUP. We treated a 75-year-old Japanese man with an MUP localized to the axillary lymph nodes. The primary tumor could not be identified despite available investigations. We performed radical lymph node dissection and administered postoperative adjuvant therapy using the molecular-targeted drugs dabrafenib and trametinib. Ten months after surgery, the patient experienced a favorable clinical course with no recurrence or complications from surgery or medication. The frequency and clinical characteristics of malignant melanoma differ among ethnic groups, and very few reports of MUP have been documented in East Asia.

## Introduction

Malignant melanoma is classified as a *rare cancer* in Japan [1]. Its prognosis is poor once the metastasis has occurred [2]. The standard treatment for melanoma involves resection of the primary lesions; however, 3.2% of patients present with melanoma of unknown primary origin (MUP) [3].

MUP was first defined by Das Gupta in 1963 as "melanoma discovered in subcutaneous tissues, lymph nodes (LNs), or visceral organs without a cutaneous, ocular, or mucosal primary site" [4]. Additionally, four exclusion criteria were established, yet only 16% of reported cases were diagnosed according to the definition; most others were considered MUP based on physical examination alone [3]. Most MUP reports are from countries with a predominantly Caucasian population [3]. In East Asia, only 5 cases of MUP confined to LNs have been reported in PubMed.

Here, we report a rare case of MUP localized to the axillary LNs. It was treated with LN dissection and molecular-targeted agents and showed a good course without recurrence.

## Case presentation

A 75-year-old Japanese man presented to our hospital with an asymptomatic 4-cm axillary mass, which had been gradually increasing in size for one month. He had a history of hypertension, epilepsy, and herniated disc surgery. He was prescribed antiepileptic and antihypertensive medications. He had no family history of malignancy, no previous treatment for skin lesions, such as lentigines or pigmented macules, and no trauma-related skin defects.

**Figure 1:**
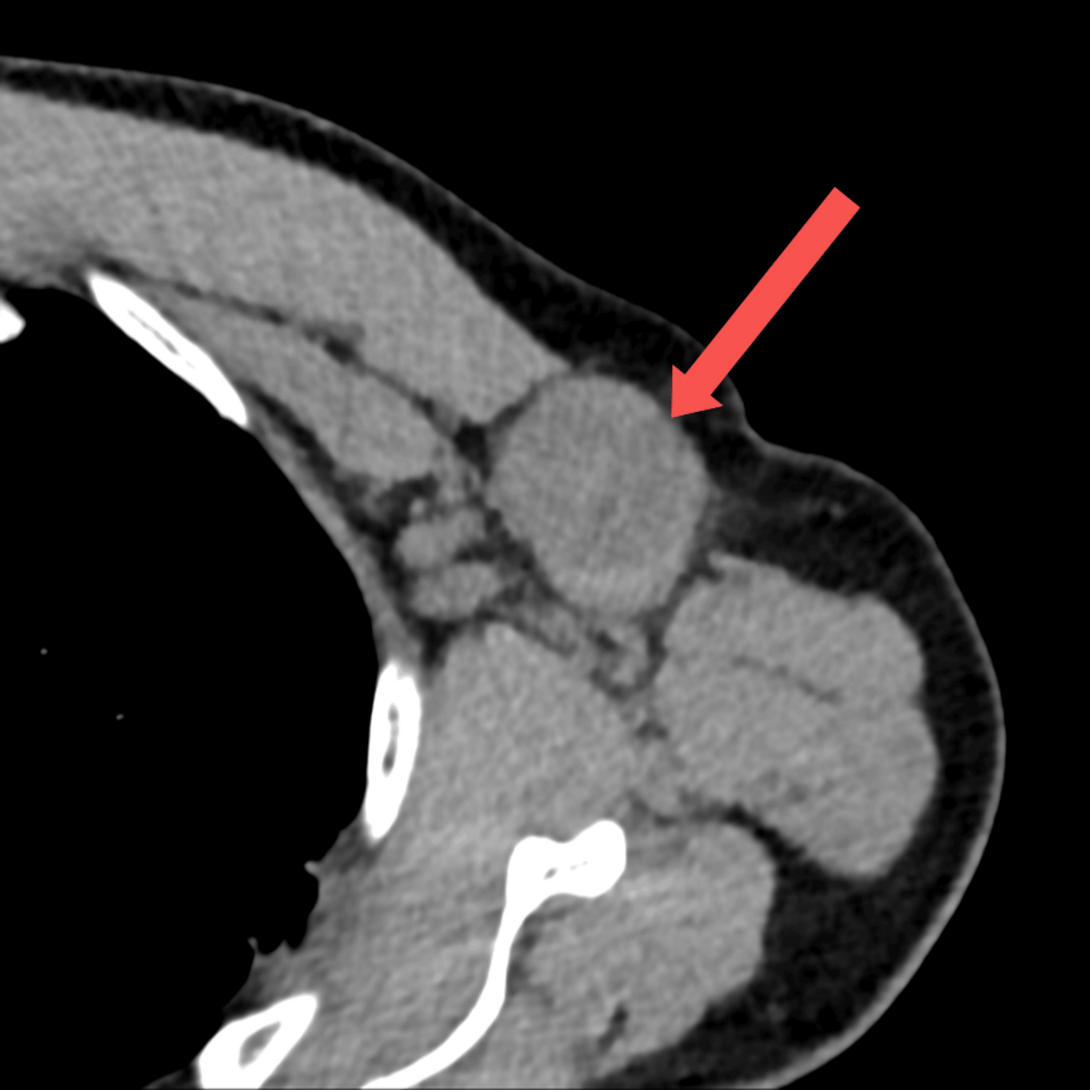
Preoperative computed tomography image of the left axillary mass.

**Figure 2:**
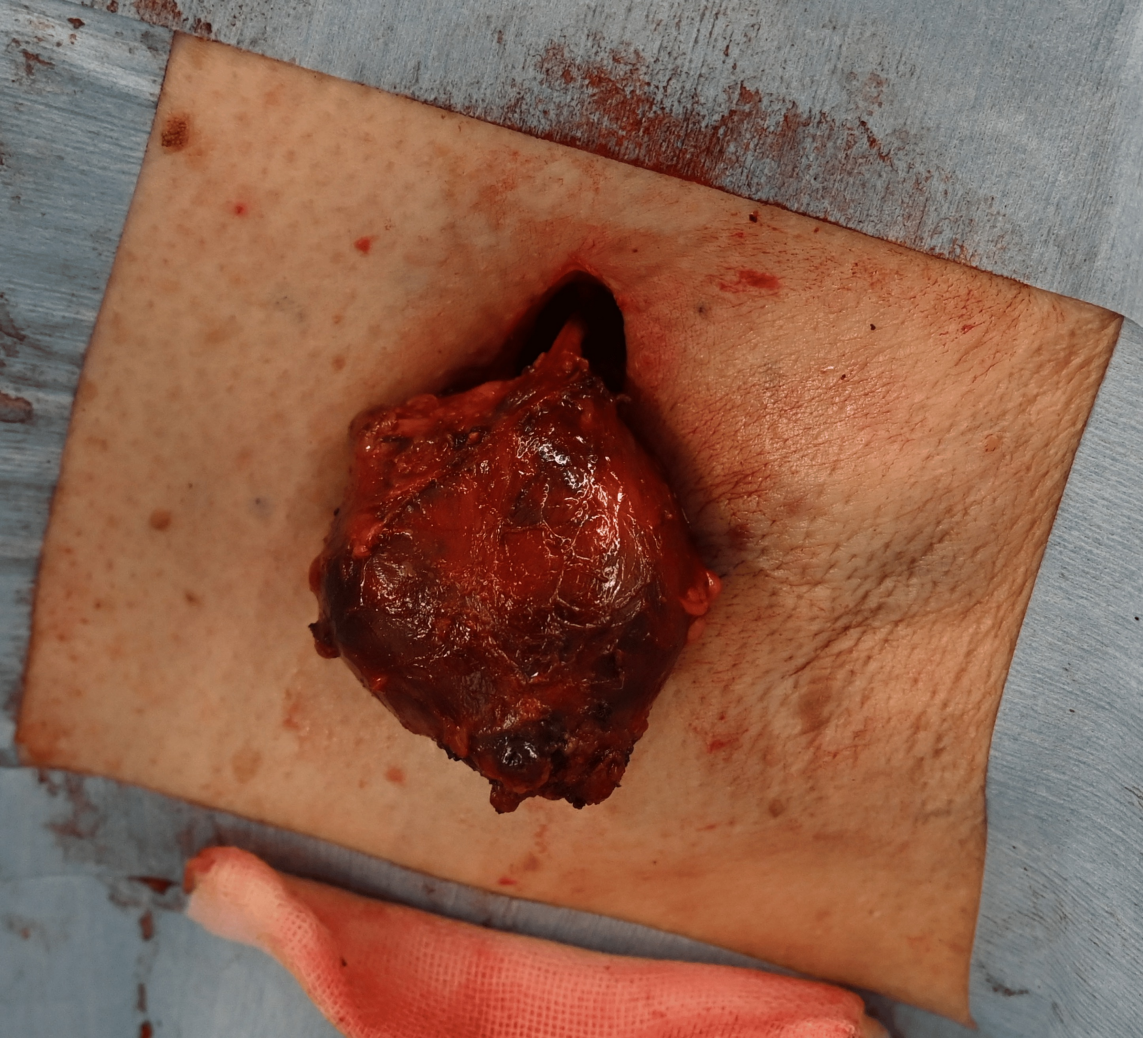
A lymph node was resected.

**Figure 3:**
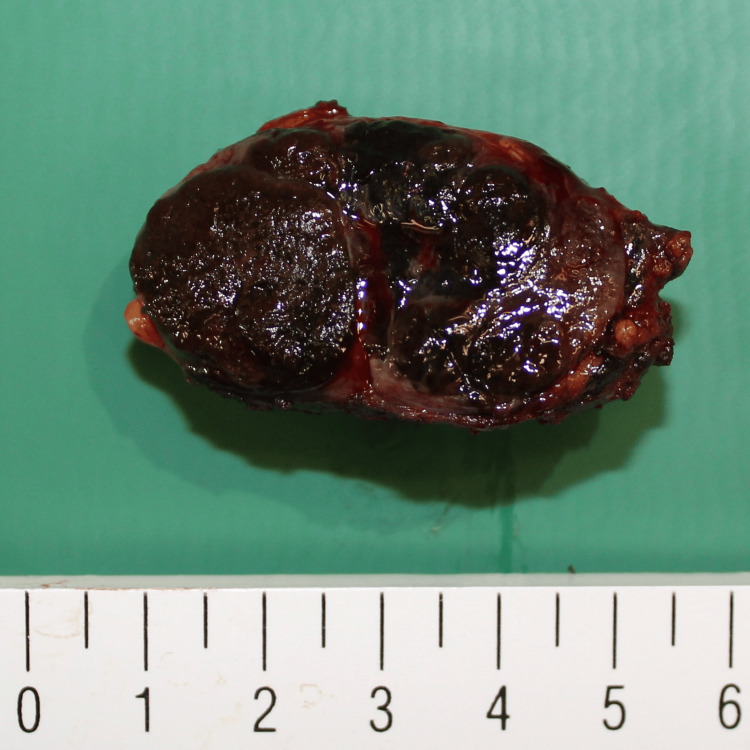
Almost all cross-sections of the lymph nodes were black.

Computed tomography (CT) revealed a 42-mm LN and three LNs > 1 cm in the left axilla (Figure 1). We suspected malignant lymphoma and removed the largest LN to determine the diagnosis and treatment strategy (Figure 2). The cross-sections of the LN were almost entirely black (Figure 3).

**Figure 4:**
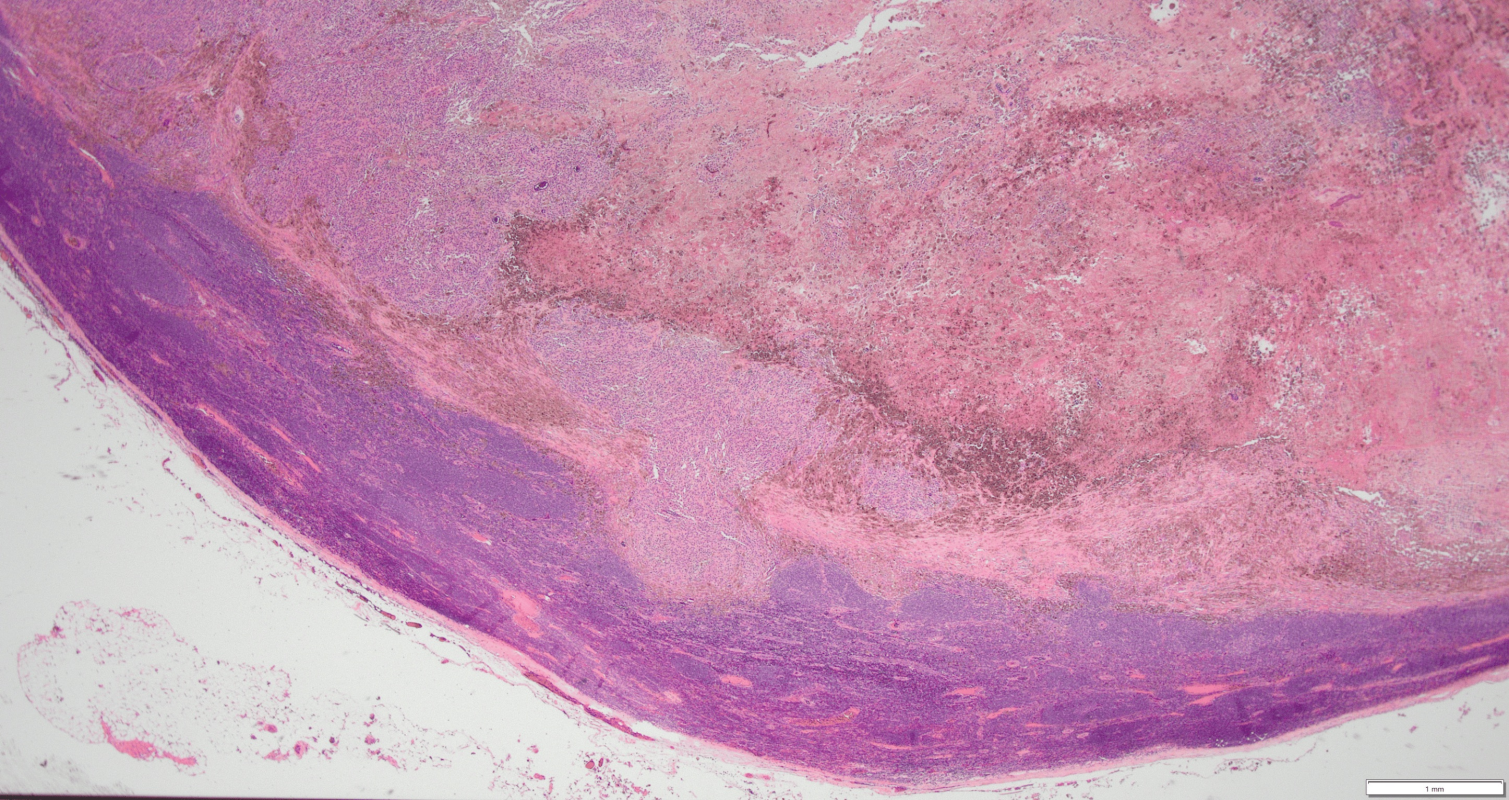
Pathological examination revealed a sheet-like growth of tumor cells. Numerous tumor cells contained melanin, and the tumor exhibited areas of necrosis (hematoxylin-eosin, x12.5).

**Figure 5:**
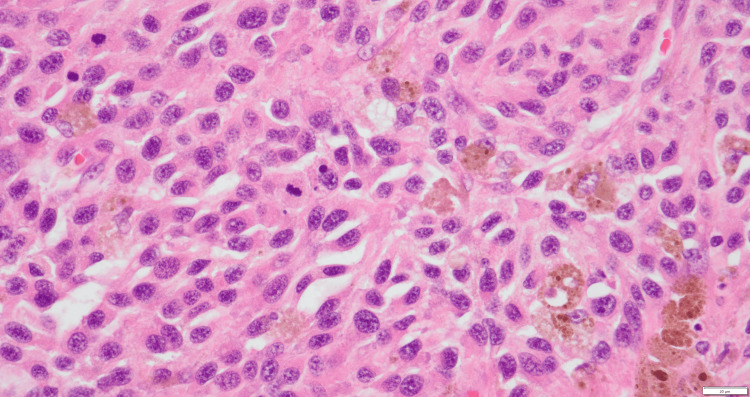
Atypical nuclei and enlarged nucleoli were observed, along with melanin granules (hematoxylin-eosin, x400).

**Figure 6:**
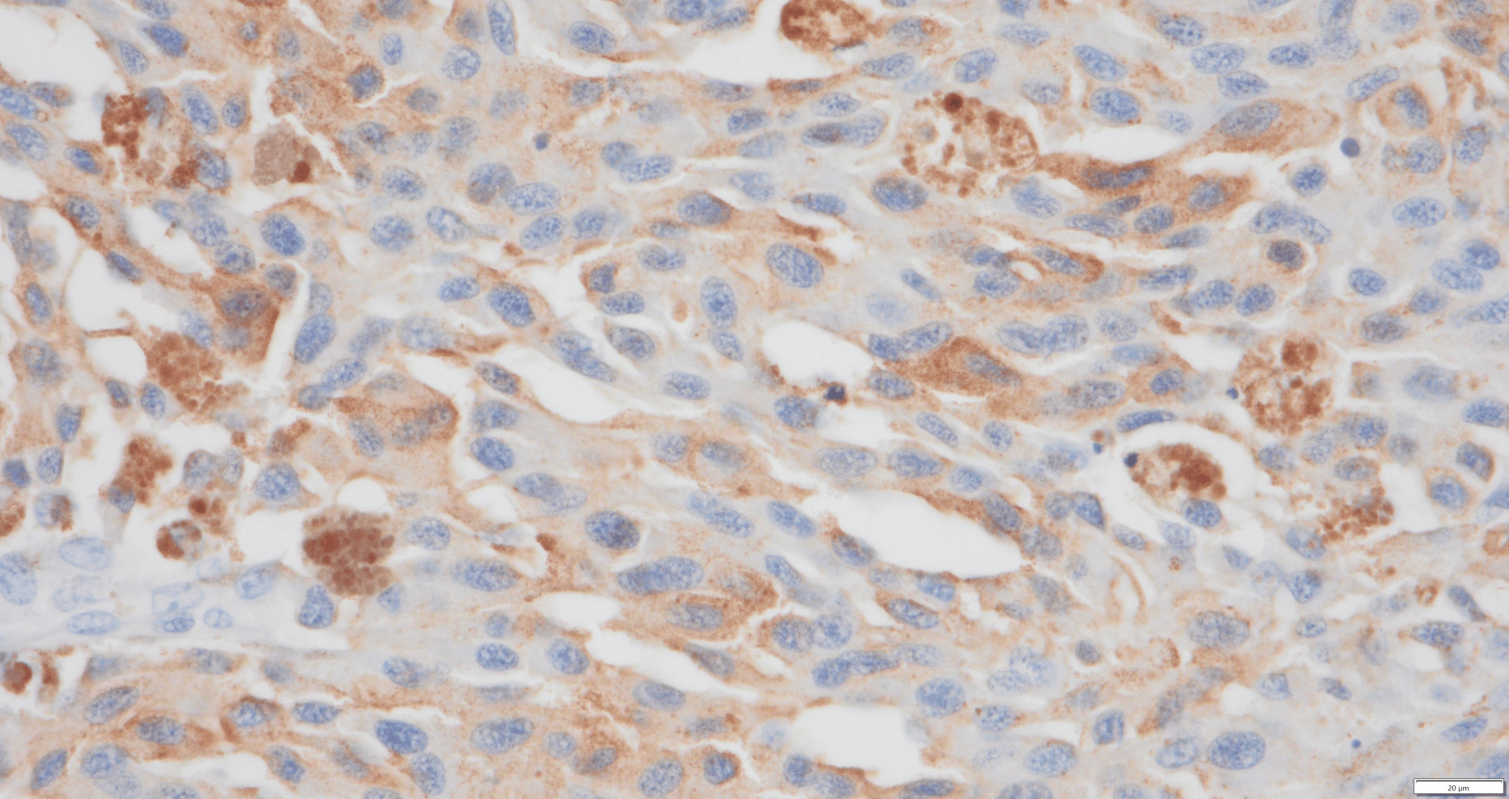
Immunohistochemical staining for S-100 was positive (x400).

**Figure 7:**
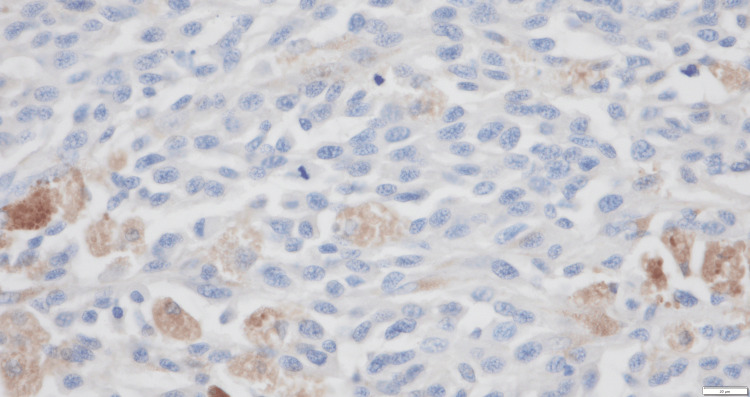
Immunohistochemical staining for Human Melanoma Black-45 (HMB-45) was positive (x400).

Pathological examination revealed a sheet-like growth of tumor cells. Numerous tumor cells contained melanin, and the tumor exhibited areas of necrosis (Figure 4). Atypical nuclei and enlarged nucleoli were observed, with melanin granules (Figure 5). Immunohistochemistry was positive for S-100 (Figure 6) and Human Melanoma Black-45 (Figure 7) and negative for leukocyte common antigen and anti-cytokeratin monoclonal antibodies 1 and 3.

The v-Raf murine sarcoma viral oncogene homolog B (BRAF) gene mutation was also detected. These findings led to the diagnosis of malignant melanoma. Tumor markers were normal, with a neuron-specific enolase level of 15.5 ng/mL and 5-S-cysteinyldopa 5.4 nmol/L.

A head-to-toe skin examination, including the pubic region, using dermoscopy was performed by two dermatologists and three plastic surgeons to identify the primary tumor. Overall, four skin lesions with either irregular streaks or dots in which melanoma could not be ruled out were biopsied, and all were diagnosed as nevi on pathological examination. There was no evidence of vitiligo. The palate-pigmented mucosa biopsy revealed metallosis. An ophthalmological examination did not reveal any pigmented lesions in the choroid. No primary lesion was observed following upper and lower gastrointestinal endoscopy. An otolaryngologist's endoscopy of the oral and nasal cavity, larynx, pharynx, and external auditory canal otoscopy did not reveal any pigmented lesions. Positron emission tomography (PET)/CT showed no abnormalities except in the axillary LN. Magnetic resonance imaging of the head revealed no neoplastic lesions.

**Figure 8:**
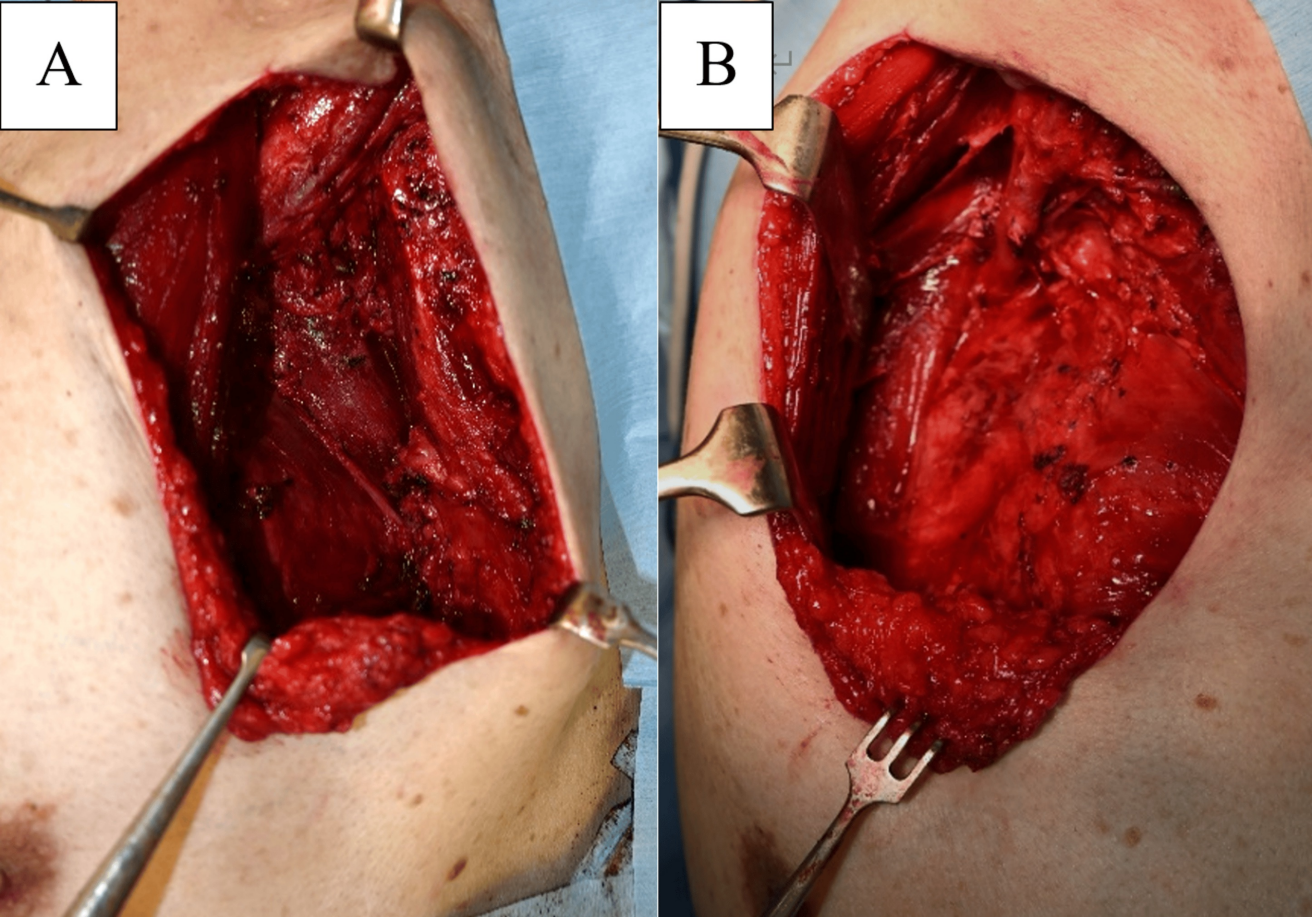
(A) The axillary lymph nodes were dissected, and (B) the subpectoral and infraclavicular lymph nodes were dissected.

Based on these results, we diagnosed MUP localized to the axillary LN and performed left axillary LN dissection under general anesthesia (Figures 8A-8B).

Five LNs were metastatic in the axilla; however, no metastasis was found in the subpectoral, interpectoral, or infraclavicular LNs. Therefore, the patient's pathological stage was determined to be IIIC (T0N3bM0) (American Joint Committee on Cancer, 8th edition [5]). The wounds healed postoperatively without complications.

**Figure 9:**
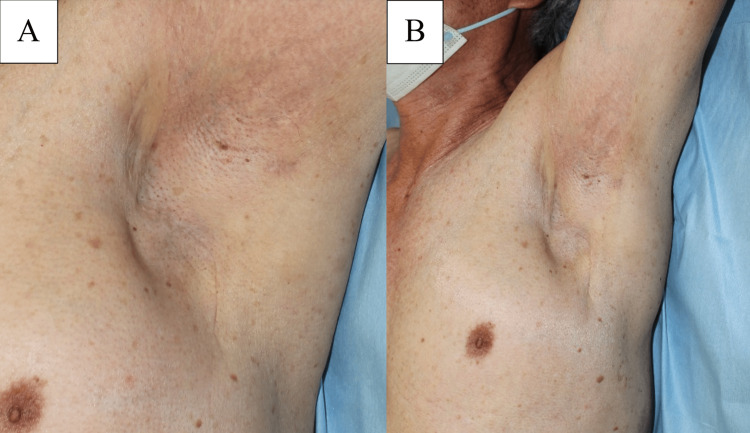
(A) Postoperative findings: no recurrence was observed at 10 months; (B) the patient continued to demonstrate a good range of motion of the shoulder.

The patient received postoperative adjuvant therapy with a combination of oral dabrafenib (150 mg twice daily) and trametinib (2 mg once daily), due to the positive BRAF mutation. Adjuvant therapy was scheduled to continue for 1 year. Ten months postoperatively, there was no recurrence in the axilla or on total skin surface examination (Figures 9A-9B).

Additionally, there were no issues with shoulder range of motion or lymphedema.

## Discussion

Because the Das Gupta definition does not reflect advances in current imaging technology, Sirvan et al. and Kamposioras et al. proposed additional criteria for diagnosing MUP [3,6].

In contrast to many previous MUP cases that failed to meet even the Das Gupta definition [3], our case fulfills all criteria, including the newly introduced ones. We diagnosed this MUP case confidently using these factors.

With the widespread use of CT in the 1980s, the incidence of MUPs decreased from 5.1% to 2.7% [3] and is expected to decline further with the widespread use of PET-CT. However, to date, no report has verified this. Ocular and mucosal melanomas account for 5.5% and 1.3% of melanomas, respectively [7]. Fundus examination and endoscopy for MUP diagnosis further reduce their frequency because they cannot be diagnosed solely by skin examination and are invisible on imaging until they have progressed.

In an extensive Japanese survey, the incidence of MUPs was reported to be 1.8% in 2011-2013 and 2.2% in 2005-2017 [1,2], less than those reported in Caucasian countries. This can be attributed to Japan's universal health insurance system, which allows for performing many tests inexpensively, and the high penetration of CT, PET/CT, and other testing equipment [8]. This may also be influenced by the fact that the standard histology of Japanese differs from that of Caucasians [2].

There are different MUP patterns; the primary lesion may be small, inconspicuous, and undetected, or it may have been resolved by innate immunity, leaving only metastases [9], or it may be ectopic nevus cells in the LNs or internal organs [10].

MUP with LN metastasis alone is less common (24.1%) [3]. It is more common in men and in the axilla, which was also observed in this case. However, the tendency for MUP to occur in individuals in their 40s and 50s does not apply to this case [3].

Owing to the paucity of MUPs localized to LNs [3], the fact that melanoma is less common in East Asian individuals [11], and the low MUP frequency in East Asia [1,2], we could find only five case reports of East Asian MUPs localized to LNs in PubMed.

Kamposioras et al. [3] conducted a systematic review of 41 peer-reviewed articles and one abstract on MUP reported over the past 89 years. However, all of these studies were from countries predominantly inhabited by Caucasian populations [3]. Compared to non-Hispanic Caucasians, East Asians have a lower proportion of superficial spreading melanoma and a higher proportion of acral lentiginous melanoma, which is associated with a poor prognosis [2,12-14]. Additionally, melanoma in East Asians is more commonly found in the lower extremities rather than the upper extremities, and it has a lower response rate to immune checkpoint inhibitors [2,12]. These differences are also expected in MUP, suggesting that the results of the systematic review by Kamposioras et al. [3] may not apply to MUP in East Asians.

Surgery is the first-line treatment for resectable malignant melanoma. In MUP, surgery may be curative in stage III cases confined to regional LNs or subcutaneous tissue. The five-year postoperative survival rate is more extended for stage III MUP versus melanoma of known primary origin (MKP) [15]. This is due to the theory that innate immunity is strong enough to allow spontaneous regression and a molecular difference exists between MKP and MUP [16,17].

In the current case, we determined that the lesion was confined to the axilla because we had performed all the tests possible and were able to decide to perform surgery. However, as there have been reports of primary lesions being discovered many years after the MUP diagnosis [18], we will continually examine the entire body for skin and imaging evaluations, carefully and regularly.

We also used BRAF and mitogen-activated protein kinase (MEK) inhibitors as postoperative adjuvant therapy for stage III melanoma, as Kamposioras et al. recommended that MUP be treated commensurately with the stage of MKP [3]. However, MUP was excluded in a trial showing the efficacy of postoperative adjuvant therapy with BRAF MEK inhibitors in stage III [19], and a report by Verber et al. showed that novel therapies (immune checkpoint inhibitors and molecular-targeted agents) were effective for stage IV MUP but failed to demonstrate prolonged overall survival in stage III patients [20]. Thus, MUP drug therapy is yet to be established, and further research is needed.

Finally, the rarity of MUP in East Asia makes large-scale clinical trials challenging. Considering the rarity of MUP, especially when confined to LNs, further case reports and series should be collected to establish treatment guidelines.

## Conclusions

We report a case of MUP in axillary LNs successfully treated with LN dissection and molecular-targeted therapy after a careful primary lesion search to rule out its presence. As a standard of MUP care is yet to be established, further accumulation of cases is required.
